# Improving the Odds in Advanced Breast Cancer With Combination Immunotherapy: Stepwise Addition of Vaccine, Immune Checkpoint Inhibitor, Chemotherapy, and HDAC Inhibitor in Advanced Stage Breast Cancer

**DOI:** 10.3389/fonc.2020.581801

**Published:** 2021-03-05

**Authors:** Margaret E. Gatti-Mays, Sofia R. Gameiro, Yohei Ozawa, Karin M. Knudson, Kristin C. Hicks, Claudia Palena, Lisa M. Cordes, Seth M. Steinberg, Deneise Francis, Fatima Karzai, Stanley Lipkowitz, Renee N. Donahue, Caroline Jochems, Jeffrey Schlom, James L. Gulley

**Affiliations:** ^1^Laboratory of Tumor Immunology and Biology, Center for Cancer Research, National Cancer Institute, National Institutes of Health, Bethesda, MD, United States; ^2^Genitourinary Malignancies Branch, Center for Cancer Research, National Cancer Institute, National Institutes of Health, Bethesda, MD, United States; ^3^Biostatistics and Data Management Section, Center for Cancer Research, National Cancer Institute, National Institutes of Health, Bethesda, MD, United States; ^4^Women’s Malignancies Branch, Center for Cancer Research, National Cancer Institute, National Institutes of Health, Bethesda, MD, United States

**Keywords:** metastatic breast cancer, bintrafusp alfa, entinostat, BN-Brachyury, TGF-β

## Abstract

Breast tumors commonly harbor low mutational burden, low PD-L1 expression, defective antigen processing/presentation, and an immunosuppressive tumor microenvironment (TME). In a malignancy mostly refractory to checkpoint blockade, there is an unmet clinical need for novel combination approaches that increase tumor immune infiltration and tumor control. Preclinical data have guided the development of this clinical trial combining 1) BN-Brachyury (a poxvirus vaccine platform encoding the tumor associated antigen brachyury), 2) bintrafusp alfa (a bifunctional protein composed of the extracellular domain of the TGF-βRII receptor (TGFβ “trap”) fused to a human IgG1 anti-PD-L1), 3), entinostat (a class I histone deacetylase inhibitor), and 4) T-DM1 (ado-trastuzumab emtansine, a standard of care antibody-drug conjugate targeting HER2). We hypothesize that this tetratherapy will induce a robust immune response against HER2^+^ breast cancer with improved response rates through 1) expanding tumor antigen-specific effector T cells, natural killer cells, and immunostimulatory dendritic cells, 2) improving antigen presentation, and 3) decreasing inhibitory cytokines, regulatory T cells, and myeloid-derived suppressor cells. In an orthotopic HER2^+^ murine breast cancer model, tetratherapy induced high levels of antigen-specific T cell responses, tumor CD8^+^ T cell/Treg ratio, and augmented the presence of IFNγ- or TNFα-producing CD8^+^ T cells and IFNγ/TNFα bifunctional CD8^+^ T cells with increased cytokine production. Similar effects were observed in tumor CD4^+^ effector T cells. Based on this data, a phase 1b clinical trial evaluating the stepwise addition of BN-Brachyury, bintrafusp alfa, T-DM1 and entinostat in advanced breast cancer was designed. Arm 1 (TNBC) receives BN-Brachyury + bintrafusp alfa. Arm 2 (HER2^+^) receives T-DM1 + BN-Brachyury + bintrafusp alfa. After safety is established in Arm 2, Arm 3 (HER2^+^) will receive T-DM1 + BN-Brachyury + bintrafusp alfa + entinostat. Reimaging will occur every 2 cycles (1 cycle = 21 days). Arms 2 and 3 undergo research biopsies at baseline and after 2 cycles to evaluate changes within the TME. Peripheral immune responses will be evaluated. Co-primary objectives are response rate and safety. All arms employ a safety assessment in the initial six patients and a 2-stage Simon design for clinical efficacy (Arm 1 if ≥ three responses of eight then expand to 13 patients; Arms 2 and 3 if ≥ four responses of 14 then expand to 19 patients per arm). Secondary objectives include progression-free survival and changes in tumor infiltrating lymphocytes. Exploratory analyses include changes in peripheral immune cells and cytokines. To our knowledge, the combination of a vaccine, an anti-PD-L1 antibody, entinostat, and T-DM1 has not been previously evaluated in the preclinical or clinical setting. This trial (NCT04296942) is open at the National Cancer Institute (Bethesda, MD).

## Introduction

Breast cancer has historically been considered immunologically cold with most tumors having a relatively low mutational burden [mean 1.63 mutations per megabase (1)] and low programmed death-ligand 1 (PD-L1) expression (2, 3). In addition, it is hypothesized that the intrinsic resistance to immunotherapy observed in breast cancer may be due to low neoantigen levels, defective antigen presentation, and the presence of transforming growth factor beta (TGF-β) and other immunosuppressive signals in the tumor microenvironment (TME), collectively promoting exclusion of effector T cells and natural killer (NK) cells from the tumor (4–7). Together, these defects create an immunosuppressive TME that impedes the generation of an effective anti-tumor immune response as is reflected by the modest results observed with immune checkpoint blockade (ICB) monotherapy in breast cancer (3, 8, 9).

The FDA recently granted the first approved indication for ICB use in breast cancer, with the use of the anti-PD-L1 antibody atezolizumab in combination with nab-paclitaxel for patients with PD-L1^+^ triple-negative breast cancer (TNBC) (10). In this new era, multimodal therapies may help shift the TME to a more immunopermissive environment, thus allowing proper engagement of the immune system to successfully eradicate the tumor.

Here, we present preclinical data and the resulting innovative clinical trial design for a phase 1b clinical trial that will evaluate safety and efficacy of combination immunotherapy through the stepwise addition of Bavarian Nordic (BN)-Brachyury, bintrafusp alfa, T-DM1, and entinostat in advanced breast cancer.

## Rationale for TetraTherapy Combination in Advanced Breast Cancer

### BN-Brachyury

BN-Brachyury vaccine is a recombinant poxvirus vaccine against the transcription factor brachyury, a tumor-associated antigen that plays an important role in the epithelial-to-mesenchymal transition in breast cancer (11–13). BN-Brachyury is comprised of two replication incompetent recombinant viral vaccines, i.e., modified vaccinia Ankara (MVA-BN-Brachyury; prime) and fowlpox (FPV-Brachyury; boost). Clinical data with various brachyury vaccines have consistently demonstrated generation of a brachyury-specific T cell response in breast cancer patients (14, 15). A recently completed phase 1 study of BN-Brachyury prime-boost vaccines showed the vaccine was well tolerated and generated brachyury-specific immune responses in patients with advanced solid tumors (16).

### Bintrafusp Alfa

Bintrafusp alfa (*previously designated M7824*) is a novel bifunctional fusion protein composed of a monoclonal antibody against human PD-L1 fused to the soluble extracellular domain of human TGF-β receptor II (TGF-βRII), which functions as a TGF-β “trap” (17–19). Preclinically, bintrafusp alfa has synergized with vaccines (20). Phase 1 studies have demonstrated an acceptable safety profile and have suggested some signs of clinical benefit (21, 22). A small pilot study of bintrafusp alfa in TNBC demonstrated a response rate of 9.1%, which is comparable to other ICB in unselected TNBC (22). Multiple phase II studies are ongoing with bintrafusp alfa in breast cancer (NCT03579472, NCT03524170) [clinicaltrials.gov].

### Entinostat

Entinostat is a class 1 histone deacetylase inhibitor (HDACi) that has been shown to increase sensitivity of breast cancer cells to antigen-specific CD8^+^ T cell mediated lysis *in vitro* (23). This immunogenic modulation was observed against a broad range of tumor-associated antigens (TAAs) including brachyury and was associated with increased expression of antigen processing machinery and tumor immune recognition. HDACi have been shown to restore expression of MHC class I proteins and antigen processing and presentation molecules (23, 24). Entinostat exposure also increased the sensitivity of breast cancer cells (MDA-MB-231) to NK cell-mediated attack through direct lysis and *via* anti-PD-L1-mediated antibody-dependent cell-mediated cytotoxicity (ADCC) (25). These effects were associated with increased expression of NK ligands on tumor cells and augmented NK activation and cytolytic function upon entinostat exposure *in vitro*. Furthermore, entinostat increased the expression of human PD-L1 in PC-3 (prostate) carcinoma xenografts *in vivo* (25). Others have also shown upregulation of PD-L1 on tumors with entinostat treatment (26), which has been attributed to the epigenetic modulation observed with HDACi (24). In addition, the combination of entinostat with nivolumab and ipilimumab in patients with advanced HER2 negative breast cancers demonstrated altered myeloid-derived suppressor cell (MDSC) signaling pathway and increased immune infiltration (27). Preliminary results of the ENCORE-601 trial involving entinostat with pembrolizumab in checkpoint refractory non-small cell lung cancer (NSCLC) demonstrated clinical efficacy in multiple patients regardless of prior checkpoint treatment or PD-L1 status (28). However, despite strong preclinical evidence regarding the combination of entinostat with ICBs, trials results have been inconsistent. In ENCORE-602 (29) and ENCORE-603 (30), entinostat was combined with an anti-PD-L1 antibody in patients with advanced TNBC (ENCORE-602) or advanced epithelial ovarian cancer (ENCORE-603). The combination did not improve clinical efficacy in these populations and a higher number of grade 3 and 4 treatment related adverse events (AEs) were seen in the combination arms, with the most frequent toxicities seen being neutropenia and fatigue (29, 30). However, with dose modifications, most patients were able to continue on therapy. In addition, there were no increases in immune-related adverse events in the combination arms over the anti-PD-L1 monotherapy arm.

### T-DM1

Ado-trastuzumab emtansine (T-DM1 or Kadcycla^®^) is an antibody-drug conjugate used in the second- and third-line treatment of metastatic HER2^+^ breast cancer (HER2^+^ BC). T-DM1 activates ADCC, dendritic cell maturation, and increases tumor infiltrating lymphocytes (TILs), PD-L1 expression, and immunomodulatory cytokines (31, 32). The KATE2 trial evaluated T-DM1 +/- atezolizumab. There was no significant improvement in the primary trial endpoint of progression-free survival (PFS) in the intention to treat (ITT) population. Exploratory analysis of overall survival data in the ITT population is still maturing but an interim report at ESMO 2019 showed that the median survival had not been met in either treatment arm. In addition, survival analysis stratified by PD-L1 expression demonstrated a trend towards longer survival in PD-L1+ patients (94.3% in PD-L1+ vs 87.9% in PD-L1-) but this was not significant (HR 0.55, 95% CI 0.22 to 1.38) (33, 34).

## Preclinical Data Supporting TetraTherapy in Advanced Breast Cancer

**Figure 1:**
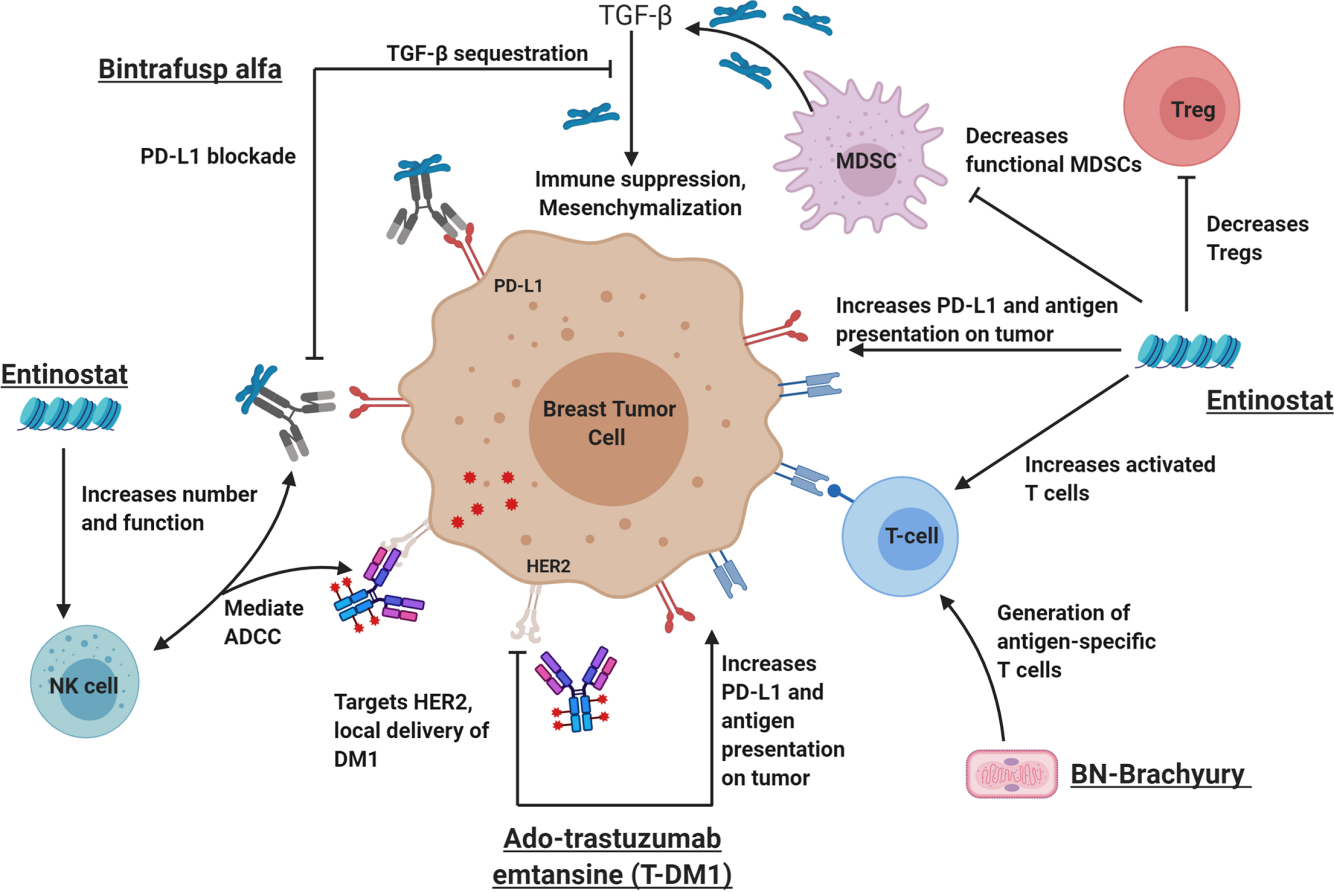
The rational combination of immunotherapy agents can augment key components of a successful anti-tumor immune response. From left to right: Bintrafusp alfa inhibits PD-L1 and sequesters TGF-β in the tumor microenvironment leading to activation of immune effector cells (including natural killer cells) and decreased tumor plasticity. Ado-trastuzumab emtansine (aka T-DM1I, Kadcyla) is an antibody drug conjugate that targets the HER2 receptor and allows the delivery of the anti-microtubule chemotherapy called DM1. Like other HER2-targeting antibodies, T-DM1 also mediates ADCC, increases antigen presentation on tumor cells and increases PD-L1 expression on the tumor and immune cells. BN-Brachyury vaccines generate brachyury-specific T cell responses in breast cancer. Brachyury is a transcription factor involved in tumor plasticity, metastasis, chemoresistance, and poor clinical outcomes in breast cancers. Entinostat is an HDACi that increases antigen presentation, increases activated T cells and decreases Tregs. In addition, entinostat is also known to decrease HER2 resistance through various mechanisms. It is hypothesized that this tetratherapy will induce a robust immune response against breast cancer with improved tumor control. ADCC, antibody-dependent cell-mediated cytotoxicity; HDACi, histone deacetylase inhibitor; MDSCs, myeloid-derived suppressor cells; NK cell, natural killer cell; PD-L1, programmed death-ligand 1; TGF-β, transforming growth factor beta. This figure was created by MG-M using BioRender.com.

Previously, Christmas et al. (35) demonstrated synergistic anti-tumor activity with the triple combination of entinostat, an anti-programmed cell death protein 1 (PD-1) antibody and a HER2-targeted antibody in HER2/neu transgenic breast cancer models, resulting in significant reduction in tumor size and improved survival. This was associated with reprogramming of granulocytic MDSCs in the TME to become less suppressive, increased CD8^+^ effector T cells, and reduced regulatory T cells (Tregs) compared to single agent HER2-targeted therapy. Based upon this preliminary data along with the known immune effects and anti-tumor activity of BN-Brachyury (14, 16, 36), entinostat (23, 25, 35, 37), bintrafusp alfa (38–40), and T-DM1 (31, 32), we developed a preclinical hypothesis that the 4-agent combination with entinostat (E), bintrafusp alfa (B), Ad-Twist vaccine (A), and T-DM1 (T) (EBAT) would provide superior anti-tumor activity and immune effects in a breast cancer model (**Figure 1**) compared to the triplet (BAT), doublet (BA) or single agents (E, T, or B). To our knowledge, the combination of a vaccine, an anti-PD-L1 antibody, entinostat, and T-DM1 has not been previously evaluated in the preclinical or clinical setting.

To test this hypothesis, we examined the antitumor effects and immune correlates of the 4-agent combination therapy in the HER-2 expressing TuBO murine breast cancer model. Of note, since TuBO cells do not express brachyury, Twist was used as a model antigen. Twist is well-expressed in TuBO tumor cells and shares functional features with brachyury in multiple aspects of tumor progression. Similar to brachyury, Twist is a transcription factor that has also been implicated in the control of tumor plasticity and as a driver of metastatic progression (11, 13, 41, 42). Overexpression of either brachyury or Twist has been associated with poor prognosis for multiple carcinomas, including breast cancer (11, 43, 44). In preclinical studies, a vaccine targeting Twist demonstrated significant anti-tumor and anti-metastatic effects as a monotherapy (45).

**Figure 2:**
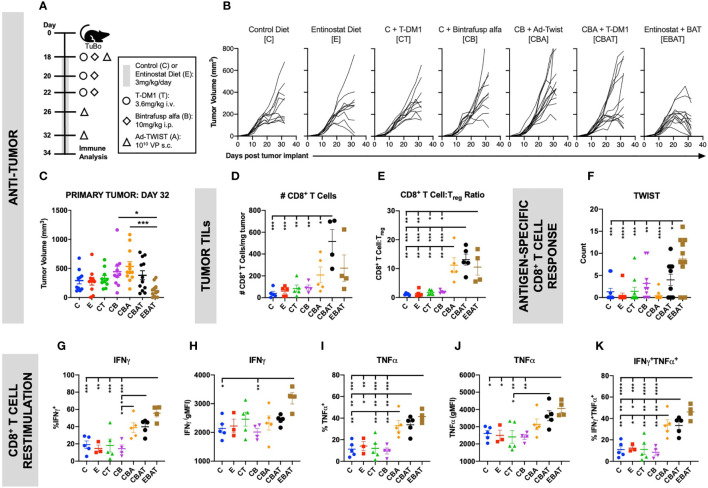
Combination therapy resulted in improved anti-tumor immune response in an orthotopic HER2^+^ murine breast cancer model. TuBo breast tumor cells (4×10^5^) were orthotopically implanted into the mammary fat pad of female Balb/c mice on day 0 and treated with either control (C) or entinostat (E) diet, T-DM1 (T), bintrafusp alfa (B), and/or Ad-Twist vaccine (A) according to the schedule and doses in **(A)**. **(B, C)** Graphs show primary tumor growth curves of individual mice in each treatment group over the entire study duration **(B)**, and at day 32 **(C)**. For **(B, C)**, data from 1 experiment, n=11–12 mice/group. **(D–K)** Two days after the last vaccination, tumor, and spleen immune cells were isolated. Graphs show the number of CD8^+^ T cells **(D)**, or the ratio of CD8^+^ T cells to T_reg_ **(E)** in the tumor. **(F)** Splenocytes were incubated with a Twist peptide pool overnight and IFNγ production was determined by ELISPOT. **(G–K)** Tumor CD8^+^ T cells were stimulated with αCD3 and αCD28 for 4 h and cytokine production was analyzed by flow cytometry. Graphs show frequency of total IFNγ^+^ **(G)**, IFNγ production on a per cell basis **(H)**, frequency of total TNFα^+^ **(I)**, TNFα production on a per cell basis **(J)**, and frequency of IFNγ/TNFα-double producing **(K)** CD44^hi^ CD8^+^ T cells. For **(D–K)**, data from 1 experiment, n=3-5 mice/group. All graphs show mean ± SEM. Data were analyzed using one-way ANOVA with Tukey’s multiple comparisons. Statistical significance was set at p <0.05. *p < 0.05, **p < 0.01, ***p < 0.001, ****p < 0.0001.

TuBo murine breast tumor cells were orthotopically implanted subcutaneously (s.c.) into the mammary fat pad of female Balb/c mice. Tumor volume was measured twice weekly. When tumors reached 200mm^3^, mice were randomized based on tumor size and treatment initiated as per the schematic (**Figure 2A**). Mice received entinostat (E) or control (C) chow. The Ad-Twist vaccine (A) was administered s.c. at a non-tumor site. Bintrafusp alfa (B) and T-DM1 (T) were administered intraperitoneally (i.p.) and intravenously (i.v.), respectively. Animals were monitored by the veterinarian staff for signs of toxicity, including weight loss, signs of pain, lethargy, or any abnormal behavior. No toxicity was reported in this study. Animals were sacrificed on day 34 and immune populations in the tumor were examined (**Figures 2D–K** and **Supplemental Figure S1**). As shown in **Figure 2**, combination therapy with entinostat, bintrafusp alfa, Ad-Twist vaccine, and T-DM1 (EBAT) induced the highest level of anti-tumor activity of all treatment groups (**Figures 2B, C**, and **Supplemental Figure S1**), with 100% of tumors regressing at day 32 (**Figure 2B** and **Supplemental Figure S1a**). While not statistically significant, there was a trend towards EBAT therapy enhancing CD8^+^ T cell tumor infiltration (**Figure 2D**), but no change in the infiltration of Tregs (**Supplemental Figure S1b**). However, the ratio of CD8^+^ T cells to CD4^+^ Tregs (**Figure 2E**) was increased, suggesting a more immunopermissive TME. Furthermore, functional analyses revealed EBAT increased the functional activity of T cells. There was a significant increase in splenic Twist-specific CD8^+^ T cell responses upon EBAT treatment compared to monotherapy, dual or triple agent regimens (**Figure 2F**). Analysis of tumor CD8^+^ T cell responses (**Figures 2G–K**) upon T-cell receptor stimulation demonstrated that EBAT treatment induced the highest proportion of CD8^+^ T cells producing IFNγ (**Figure 2G**) and TNFα (**Figure 2I**), as well as multifunctional IFNγ/TNFα double positive T cells (**Figure 2K**). Furthermore, EBAT induced a significant increase over control treatment in IFNγ (**Figure 2H**) and TNFα (**Figure 2J**) expression on a per cell basis, which was not observed with monotherapy, dual or triple agent regimens. Similar results were observed in effector CD4^+^ T cells (**Supplemental Figure S1c-g**). Collectively these results suggest that EBAT tetratherapy induces superior immune-mediated anti-tumor activity relative to monotherapy, dual or triple agent regimens in a preclinical model of HER2^+^ breast cancer.

## Clinical Trial Design

### Patient Eligibility

The BrEAsT (BN-Brachyury, Entinostat, Ado-trastuzumab emtansine, and Bintrafusp alfa) study enrolls patients (female and male) with histologically or cytologically confirmed metastatic TNBC or ER^-^/PR^-^/HER2^+^ breast cancer. In this study, hormone receptor negative is defined by immunohistochemistry (IHC) as estrogen receptor (ER) < 10%, progesterone receptor (PR) < 10%, in order to include patients with low ER^+^ tumors, where the clinical benefit of endocrine therapy is unclear (46). This relaxed definition is supported by the new 2020 American College of Pathology (ACP) guidelines (46) and by molecular analysis showing similar ER gene signature scores in tumors with ER < 1% (negative) and ER 1 to 9% (47). Furthermore, gene expression signatures in tumors with ER 1 to 9% demonstrated that 48% had gene expression signatures that were basal-like, with only 8% of these tumors being identified as luminal B (47). For Arm 1 (TNBC), HER2 negative or unamplified breast cancer is defined as IHC 0 or 1+ or IHC 2+ with FISH average HER2 copy number < 4.0 signals per cell or HER2/CEP17 < 2.0 with average HER2 copy number < 4.0 signals per cell. For Arms 2 and 3 (HER2^+^), HER2 positivity is defined as HER2 amplified by IHC 3+ or FISH average with HER2 copy number ≥ 6 signals per cell or HER2/CEP17 ≥ 2.0.

Patients must be ≥ 18 years old with an Eastern Cooperative Oncology Group performance status of ≤ 1, adequate organ function including cardiac function (Ejection Fraction ≥ 50%) and bone marrow function. All patients must have measurable disease per RECISTv1.1 and HER2^+^ patients must have biopsiable disease. At least 1 prior treatment in the metastatic setting is required. Patients with known PD-L1 positive TNBC (positive according to the SP142 assay) must have received prior treatment with atezolizumab + nab-paclitaxel. TNBC patients with ER 1%–9% must have received treatment with at least two lines of endocrine treatment (tamoxifen, aromatase inhibitor, or fulvestrant), with one line including a CDK4/6 inhibitor plus endocrine therapy for metastatic cancer and be considered endocrine therapy resistant. HER2^+^ patients must have received prior treatment for metastatic disease with a taxane, trastuzumab, and pertuzumab (THP).

There is a required treatment washout period of 3 weeks for chemotherapy, 4 weeks for radiation, anti-PD-1/-L1 therapy, and other investigational agents. Asymptomatic or brain metastases treated > 6 weeks are allowed. Well controlled human immunodeficiency virus (HIV), hepatitis B virus (HBV) or treated hepatitis C virus (HCV) is allowed. Exclusion criteria include symptomatic brain metastases or clinically significant bleeding (≤ 3 months from study entry). Patients with active autoimmune conditions requiring immunosuppression are excluded. Concurrent use of immunosuppressive drugs including therapeutic prednisone is not allowed. Due to the potential for cardiac dysfunction and myocarditis, patients with a history of myocarditis are excluded.

Baseline screening assessments include a full history and physical exam, assessment of functional status, basic laboratory evaluations (complete blood count, comprehensive metabolic panel, coagulation studies, thyroid function), viral studies (HBV, HCV and HIV), cardiac evaluation with an electrocardiogram and a 2D echocardiogram and imaging studies (CT CAP and bone scan) to confirm measurable disease.

### Study Design

**Figure 3:**
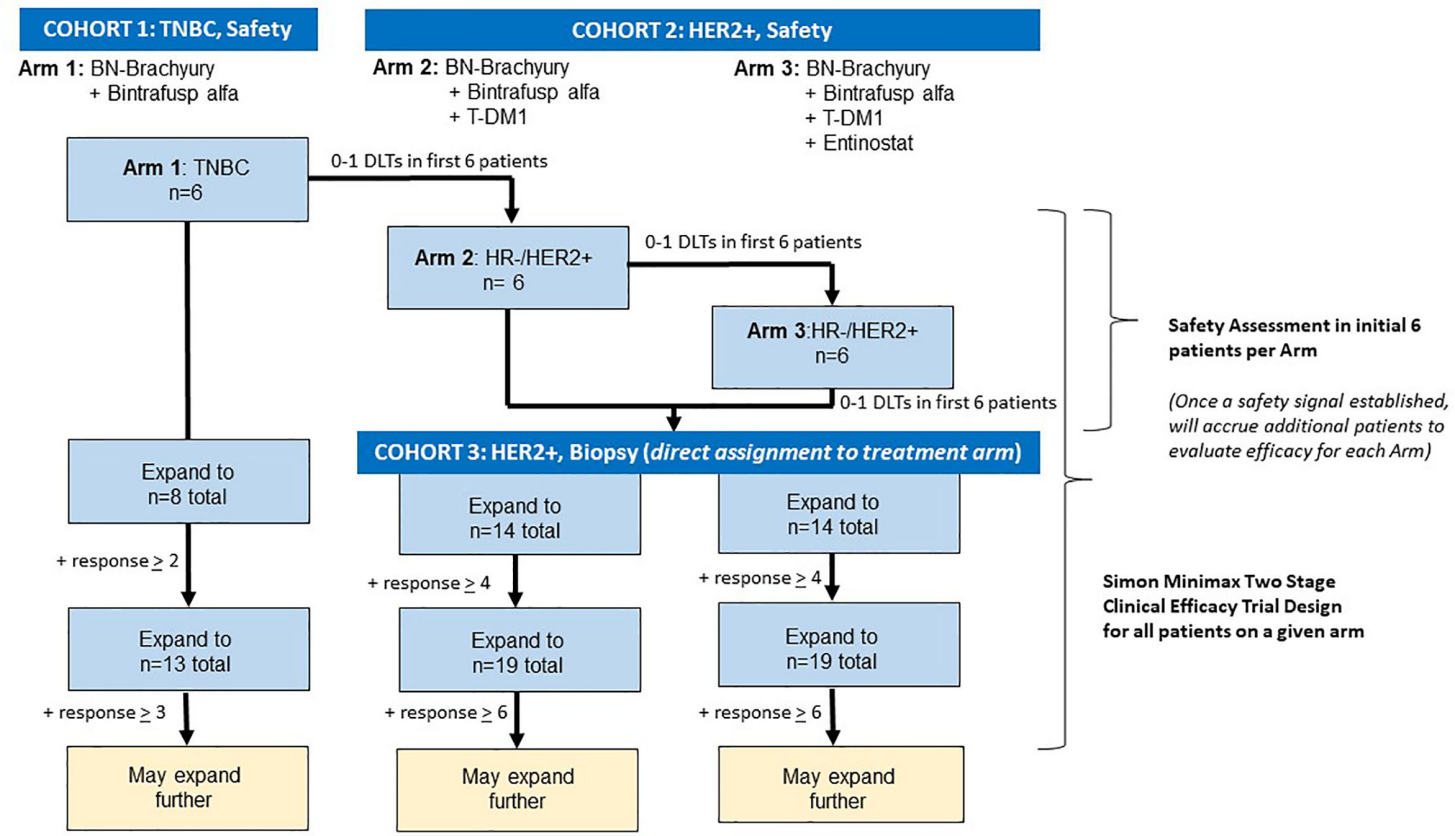
Trial schema. The BrEAsT study contains three separate, single arm phase 1b trials that evaluate the safety and efficacy of the sequential addition of immunotherapy agents with the goal of rapidly escalating to the 4-drug combination to lead to the best anti-tumor control and immune infiltration seen in the preclinical studies. Each arm starts with a safety assessment of the drug combinations with DLT assessments in the first six patients on each arm. Following the demonstration of safety, additional patients will be enrolled using a Simon 2-stage clinical efficacy design. DLT, dose limiting toxicity.

This is an open label, phase 1b trial with three arms to evaluate BN-Brachyury, entinostat, bintrafusp alfa +/- T-DM1 in advanced breast cancer (**Figure 3**). Arm 1 will evaluate BN-Brachyury and bintrafusp alfa in TNBC. Arms 2 and 3 will evaluate BN-Brachyury, bintrafusp alfa, T-DM1 +/- entinostat in ER^-^/PR^-^/HER2^+^ breast cancer. If a patient is removed from treatment, that patient will not be allowed to enroll on another study arm. Up to 51 patients will be treated on this study with an accrual ceiling set at 55 patients.

### Drug Administration

Treatment cycles are 21 days in length. Patients in Arm 1 will receive BN-Brachyury in a prime-boost fashion. The priming doses of MVA-BN-Brachyury consist of one injection in each extremity (four injections per priming dose) with each injection consisting of 2 × 10^8^ infectious units (IU) per 0.5 ml administered s.c. on day 1 of cycles 1 and 2. The boosting dose of FPV-Brachyury consists of one injection s.c. with 1 x 10^9^ IU/0.5 ml every 3 weeks until cycle 9, then every 12 weeks. Bintrafusp alfa will be administered IV every 3 weeks using a 2,400 mg flat dose. T-DM1 is given at the standard dose of 3.6 mg/kg IV every 3 weeks. Patients will self-administer entinostat 5 mg by mouth every week while on trial (**Table 1**). Dose reductions for bintrafusp alfa (1,800 mg IV q3 weeks), entinostat (3 mg po q7 days, 2 mg po q7 days) and T-DM1 (per FDA package insert) are permitted. A prophylactic dose of a 5-HT3 antagonist or dopamine blocker will be given prior to T-DM1 and patients in Arm 3 will be prescribed an oral anti-emetic to self-administer as needed due to the known gastrointestinal side effects of entinostat.

**Table 1:** Trial agents and dosing schedules*.

Agent	Manufacturer	Dose	Arms
BN-MVA-Brachyury	Bavarian Nordic	**DL1:** 4 injections SC per dose q3 weeks x 2 doses (1 injection = 2x10^8^ Inf.U/0.5 ml)	1, 2, 3
FPV-Brachyury	Bavarian Nordic	**DL1:** 1 injection SC per dose q3 weeks x 4 doses then q12 weeks (1 injection = 1x10^9^ Inf.U)	1, 2, 3
Bintrafusp alfa	EMD Serono	**DL1:** 2400mg IV q3 weeks **DL-1:**1,800 mg IV q3 weeks	1, 2, 3
T-DM1	Standard of Care	**DL1:** 3.6mg/kg IV q3 weeks **DL-1:** 3.0 mg/kg IV q3 weeks **DL-2:** 2.4 mg/kg IV q3 weeks	2, 3
Entinostat	Syndax	**DL1:** 5 mg po q 7 days **DL-1:** 3 mg po q7 days **DL-2:** 2 mg po q7 days	3

1 Cycle = 21 days; q = every.

*The stepwise addition of agents with continuous safety assessment will assist in determining any potential additive toxicities of the agent combination. Dose modifications for agents will be performed for toxicity based upon known treatment-related adverse events of each respective agent. One or more agents may be decreased or held at any time based upon high grade toxicity possibly/probably/definitely attributed to a specific trial drug or drugs. DL1, dose level 1; DL-1, dose level minus 1; DL-2, dose level minus 2.

During cycle 1, patients will return to the clinic on day 8 for a safety visit, which includes a physical exam, basic labs and repeat electrocardiogram. Starting with cycle 2, patients will be clinically assessed every 3 weeks and undergo evaluation with physical exams, functional status assessment and basic laboratory evaluations (CBC, CMP). Patients who receive T-DM1 will undergo a 2D echocardiogram every 84 days as recommended by current guidelines.

### Dose-Limiting Toxicity

Dose-limiting toxicities (DLTs) are based on the National Cancer Institute’s Common Terminology Criteria for Adverse Events (CTCAE), version 5. A DLT is defined as an adverse event or abnormal laboratory value assessed as suspected to be trial treatment related (possible, probable or definite) and unrelated to disease or disease progression that occurs within the 30 days of the first treatment. Any grade ≥3 non-hematologic, non-hepatic adverse event will be considered a DLT with the following exceptions: nausea and/or vomiting or electrolyte imbalances that persist for 48 h despite supportive care. DLTs will also include grade ≥ 3 fatigue lasting ≥ 7 days, hematologic abnormalities (grade ≥ 4 neutropenia lasting ≥ 7 days, grade ≥ 3 febrile neutropenia, grade ≥ 4 thrombocytopenia), grade ≥ 3 bleeding events, and liver abnormalities (grade ≥ 4 elevation in AST or ALT, grade ≥ 4 elevation in bilirubin).

### Endpoints

#### Safety Assessments

Up until the time the first three patients complete at least one cycle of treatment on each arm, accrual will proceed slowly (in Arm 1, no more than one patient per 6 days; in Arms 2 and 3, no more than one patient per 28 days) to more closely monitor safety, and after three patients receive at least 1 cycle of investigational agents, a safety review will be conducted before additional patients are enrolled. If there is 1 or more DLT in the first three patients on a given arm, accrual of the next three patients on the associated arm will proceed slowly with no more than one patient per 21 days (1 cycle) to continue to closely monitor safety. On the basis of the monitoring criteria, if two of the first two patients or if three patients experience DLT within the first six patients, then the trial will halt accrual and the treatment regimen may be modified. The review will include a per-patient listing of all reported AEs to date, including actions required for dosing, to more fully review the nature, frequency, severity, and timing of the events. This information combined with fewer DLTs may also result in modification of the treatment regimen. Throughout enrollment of all arms, DLTs within the 30 days of first treatment will be summarized with pre-specified criteria based on sequential boundaries to pause enrollment to more fully review safety if excessive numbers of DLTs are observed.

#### Objective Responses

Patients will undergo restaging with CT chest, abdomen and pelvis +/- bone scan every 6 weeks (2 cycles). The co-primary endpoints of this study are safety and efficacy, with efficacy being defined as an objective response in a measurable lesion as defined by RECIST version 1.1. Secondary endpoints of this study are PFS in all treatment arms and change in TILs in Arms 2 and 3.

#### Correlative Studies

Peripheral blood samples for exploratory analysis will be collected on day 1 of cycles 1, 2, 3 and 6. Peripheral blood mononuclear cells will be evaluated for changes in immune cell subsets. A flow-based assay to interrogate over 123 peripheral immune cell subsets (48, 49) will be employed to detect any changes in different phenotypes of the following: CD8^+^ T cells, CD4^+^ T cells, Tregs, dendritic cells, B cells, NK cells, NKT cells, MDSCs, and total monocytes. In addition, the generation of brachyury-specific T cells will be analyzed using a flow-based assay (14–16) that simultaneously detects antigen-specific CD8^+^ and CD4^+^ cells, and multifunctional subsets of each; the assay is also non-MHC restricted. In patients who receive entinostat, histone acetylation will be evaluated as a surrogate marker for entinostat pharmacodynamics. Plasma and serum will be evaluated for pharmacokinetics, brachyury-specific antibodies, soluble factors including sCD27 and sCD40, TGF-β levels, and cell free DNA (cfDNA). Patients with HER2^+^ breast cancer will undergo a tumor biopsy at baseline and after 2 cycles. Biopsy samples will be evaluated for changes in the immune microenvironment, HER2 expression, PD-L1 expression, tumor mutational burden, as well as epigenetic changes induced by the agent combination.

Materials and methods pertaining to preclinical studies are described in **Supplementary Materials**.

### Statistical Considerations and Plans

#### Arm 1

In similar subjects with TNBC (unselected for PD-L1 expression) who received a checkpoint inhibitor, the objective response rate (ORR) was approximately 8%–10% (50–52). Recently presented data on the use of bintrafusp alfa in TNBC demonstrated a clinical response rate of 9.1% (22). The goal is to first determine if using BN-Brachyury plus bintrafusp alfa in a small pilot arm is safe and if this combination could improve this ORR by a modest amount in advanced breast cancer. Since a HER2 targeting agent is not included in this initial pilot arm, the patient population for BN-Brachyury plus bintrafusp alfa is limited to TNBC patients. Accrual of the first three patients on trial in this arm will be slow (no more than one patient per 6 days) in order to allow for monitoring of toxicity. If there are 0–1 patients with DLTs among the six patients enrolled on this arm, accrual to this arm will continue by using a Simon optimal 2-stage phase II trial design (53) to rule out an unacceptably low partial response (PR) + complete response (CR) rate of 10% (p0 = 0.10) in favor of an improved response rate of 35% (p1 = 0.35). With 1-sided alpha=0.10 (probability of accepting a poor treatment=0.10) and beta=0.20 (probability of rejecting a good treatment=0.20), the first stage will enroll eight evaluable subjects, and if 0 to 1 of the 8 have a clinical response, then no further subjects will be accrued. If 2 or more of the first 8 subjects have a response, then accrual would continue until a total of 13 evaluable subjects have been enrolled. If there are two subjects with a response out of 13 subjects, this would be an uninterestingly low response rate and the arm would not further expand. If there were 3 or more of 13 (23.1%) who experienced a response, this would be sufficiently interesting to warrant further study in later trials. If the response rate is 10% (unacceptable level), the probability of early termination after the first stage is 81.3%.

#### Arms 2 and 3

Following determination of safety in Arm 1 (0–1 with DLTs among the first six patients enrolled), patients who are HER2^+^ will undergo an initial safety evaluation and then will be assigned in an alternating fashion to the second and third arms. Of note, due to the small number of patients and the initial safety assessment in six patients per arm, randomization is not feasible since these six initial patients will also be used in the efficacy assessment. Provided that there are 0–1 patients with DLTs among the six patients enrolled on Arm 2, this arm will be temporarily closed, and patients will be enrolled on Arm 3 for a safety evaluation. Provided that there are 0–1 patients with DLTs among the six patients enrolled on Arm 3, patients with HER2^+^ breast cancer will be directly allocated in an alternating fashion to Arms 2 and 3. In Arms 2 and 3, accrual of the first three patients in each arm will proceed slowly with no more than one patient enrolled every 28 days in order to allow for safety monitoring. If there is one or more DLTs in the first three patients on any arm, accrual of the next three patients on the associated arm will proceed slowly with no more than one patient per 21 days (one cycle) to closely monitor safety. If there are two or more patients with a DLT among the first six treated on either arm, the trial will halt accrual pending an amendment to detail how the study will proceed from that point forward.

Real world data from similar patients with metastatic HER2^+^ breast cancer who received second-line T-DM1 after first-line treatment with THP demonstrated a response rate of 18% and a duration of treatment of 4 to 5 months (54, 55). The primary objective is to determine if either arm could improve upon the 18% response rate by a modest amount. Each arm will be conducted using a Simon minimax 2-stage phase II trial design (53) to rule out an unacceptably low PR+CR rate of 18% (p0 = 0.18) in favor of an improved response rate of 40% (p1 = 0.40). With 1-sided alpha=0.10 (probability of accepting a poor treatment=0.10) and beta=0.20 (probability of rejecting a good treatment=0.20), the first stage will enroll 14 evaluable subjects in each arm, and if 0 to 3 of the 14 have a clinical response, then no further subjects will be accrued. If 4 or more of the first 14 subjects have a response in an arm, then accrual would continue until a total of 19 evaluable subjects have been enrolled in that arm. If there were 6 or more of 19 (31.6%) who experience a response, this would be sufficiently interesting to warrant further study of that combination in later trials. If accrual ends to one arm because of insufficient activity, the other arm will remain open to enroll patients directly. If the response rate is 18% (unacceptable level), the probability of termination after the first stage is 76.5% in each arm. There will be no adjustment for the multiplicity of the three arms.

## Discussion

We hypothesize that combining these four agents will lead to a robust immune response against HR^-^/HER2^+^ breast cancer with improved response rates when compared to historical controls. The immune effects of the standard of care therapy T-DM1 may be enhanced through combination with entinostat, bintrafusp alfa, and the BN-Brachyury vaccine. In a tumor that generally does not respond to checkpoint monotherapy, this combination of agents may help to augment the three key components of a successful anti-tumor immune response (3). Furthermore, the use of novel combination approaches is in keeping with the Cancer Moonshot Task Force’s mandate, which called for the use of innovative strategies to rapidly translate new agents from bench to bedside. Rational combination of immune therapies based on preclinical data is a plausible strategy to achieve this aim and is especially warranted in treating patients who have exhausted most, if not all, therapeutic options. Enhancing immunity *via* several complementary mechanisms is a promising means to produce objective responses in an ever-increasing portion of patients who may benefit from immunotherapy.

While one of the primary objectives is response rate, we acknowledge that due to small numbers, this study is not powered to fully examine clinical efficacy even if the primary endpoint of response rate is met. If the co-primary objective of response rate is met, the trial agents would likely be evaluated further in a larger phase 2 clinical trial where clinical efficacy could be better assessed with ample power.

Since the preclinical data demonstrated the best anti-tumor activity with the 4-agent combination, this study was designed to allow for rapid escalation to the 4-agent regimen. However, since these agents have not been used in combination, we are required to demonstrate safety of the agent combinations while assessing the potential clinical impact of the respective agents. A Simon optimal design was used in Arm 1 (TNBC; bintrafusp alfa + BN-Brachyury) in order to minimize the sample size needed since preclinical data did not suggest significant improvement in tumor control with this doublet. A Simon minimax design was used in Arms 2 (HER2; bintrafusp alfa + BN-Brachyury + T-DM1) and 3 (HER2; bintrafusp alfa + BN-Brachyury + T-DM1 + entinostat) due to the need to generate informative data on clinical efficacy while limiting the number of patients who would be exposed to these agents in the event there is toxicity or even decreased efficacy of the active agents. Furthermore, due to the time it took to develop this novel trial and for the trial to progress through all of the scientific and regulatory assessments, statistics were based on the data available at the conception of the trial concept in 2017 and 2018. While more recently released response rates from the KATE2 study demonstrated ORR of around 40%–45% in both the T-DM1 arm and the T-DM1+atezolizumab arm, only half of the patients in the KATE2 trial had received prior pertuzumab (33). The specified thresholds in the BrEAsT trial to proceed to a phase 2 trial are not significantly different from the response rates documented in KATE2 and since prior treatment with pertuzumab is required, we would expect the ORR to be slightly lower than the T-DM1 arm from the KATE2 trial as has been documented (54, 55). If one or more arms of the trial were to advance to a phase 2 clinical trial, a more rigorous threshold for clinical efficacy would be employed as is the case with transition of most early clinical trials to larger phase 2 clinical trials.

Preclinical data presented here support this combination of agents and show that tetratherapy increases the functionality of CD4^+^ and CD8^+^ T cells in the TME, which is associated with augmented anti-tumor efficacy relative to the triplet, doublet or singlets. This trial design in which the safety and efficacy of various combinations of immunotherapy agents are able to be evaluated relatively quickly is just one in a series of quick efficacy seeking trials (QuEST) that are being conducted at the National Cancer Institute, National Institutes of Health (Bethesda, MD) (56). The BrEAsT trial is now open and accruing patients at the Center for Cancer Research at the National Cancer Institute, National Institutes of Health (NCT04296942).

## Data Availability Statement

The raw data supporting the conclusions of this article will be made available by the authors, without undue reservation.

## Ethics Statement

The animal study was reviewed and approved by the National Institutes of Health (NIH) Intramural Animal Care and Use Committee.

## Author Contributions

The study hypothesis was designed primarily by MG-M, SRG, LMC, SS, SL, JS, and JLG. Preclinical data was performed by SG, YO, KK, KH, CP, RD, and CJ. All authors contributed to the design and drafting of the clinical trial protocol described in this manuscript. All authors contributed to the article and approved the submitted version.

## Funding

This research was supported by the Intramural Research Program of the Center for Cancer Research, the National Cancer Institute, the National Institutes of Health, as well as through the Cooperative Research and Development Agreements (CRADAs) the National Cancer Institute has with Bavarian Nordic, EMD Serono, and Syndax.

## Conflict of Interest

CP is an inventor in NIH patents related to brachyury.

The remaining authors declare that the research was conducted in the absence of any commercial or financial relationships that could be construed as a potential conflict of interest.

## Abbreviations

ACP, American College of Pathology; ADCC, Antibody-dependent cellular cytotoxicity; AE, Adverse event; BrEAsT, BN-Brachyury, Entinostat, Ado-trastuzumab Emtansine and M7824 in Advanced Stage Breast Cancer; cfDNA, Cell free DNA; CR, Complete response; CTCAE, Common Terminology Criteria for Adverse Events; DLT, Dose limiting toxicity; EBAT, entinostat, Bintrafusp alfa, Ad-Twist vaccine, and T-DM1; ER, Estrogen receptor; FISH, Fluorescence in situ hybridization; FPV, Fowlpox virus; HBV, hepatitis B virus; HDACi, Histone deacetylase inhibitor; HCV, hepatitis C virus; HIV, human immunodeficiency virus; ICB, Immune checkpoint blockade; IHC, Immunohistochemistry; i.p., Intraperitoneally; ITT, Intention to treat; i.v., Intravenously; MDSC, Myeloid-derived suppressor cell; MVA, Modified vaccinia Ankara; NK, Natural killer; NSCLC, Non-small cell lung cancer; ORR, Objective response rate; PD-1, Programmed cell death protein 1; PD-L1, Programmed death ligand 1; PFS, Progression-free survival; PR, Partial response; PR, Progesterone receptor; s.c., Subcutaneously; TGF-β, Transforming growth factor beta; TIL, Tumor infiltrating lymphocyte; TME, Tumor microenvironment; TNBC, Triple-negative breast cancer; Tregs, Regulatory T cells.
