# Supplementary material for: Improving the Odds in Advanced Breast Cancer With Combination Immunotherapy: Stepwise Addition of Vaccine, Immune Checkpoint Inhibitor, Chemotherapy, and HDAC Inhibitor in Advanced Stage Breast Cancer
Source: Front Oncol. 2021 Mar 5;10:581801. doi: 10.3389/fonc.2020.581801 (PMC7977003; doi:10.3389/fonc.2020.581801)
Supplement: Supplementary file 1 [file Presentation_1.pdf]

Improving the Odds in Advanced Breast Cancer with Combination Immunotherapy: Stepwise Addition of Vaccine, Immune Checkpoint Inhibitor, Chemotherapy and HDAC Inhibitor in Advanced Stage Breast Cancer

Margaret E. Gatti-Mays[1], Sofia R. Gameiro[1], Yohei Ozawa[1], Karin M. Knudson[1], Kristin C. Hicks[1], Claudia Palena[1], Lisa M. Cordes[2], Seth M. Steinberg[3], Deneise Francis[2], Fatima Karzai[2], Stanley Lipkowitz[4], Renee N. Donahue[1], Caroline Jochems[1], Jeffrey Schlom[1] and James L. Gulley*[2]

1 Supplementary Material

1.1 Animal Treatment and Monitoring

Six- to 10-week old female Balb/c mice were obtained from the NCI Frederick Cancer Research Facility and maintained under specific pathogen-free conditions in accordance with the Association for Assessment and Accreditation of Laboratory Animal Care (AAALAC) guidelines. All studies were approved by the NIH Intramural Animal Care and Use Committee (IACUC). Animals were monitored by the veterinary staff for signs of toxicity, including weight loss. No toxicity was reported in this study.

1.2 Murine tumor cell lines and tumor studies

TuBo breast carcinoma cells were kindly provided by Dr. Brooke Emerling, Cornell University, and cultured as previously described.[1] Cells were confirmed *mycoplasma* free by MycoAlert Mycoplasma Detection Kit (Lonza), and used at low passage number. For anti-tumor studies, TuBo tumor cells (4×10^5, s.c.) were orthotopically implanted into the mammary fat pad of female Balb/c mice on day 0. Mice were randomized based on tumor size and treatment initiated when tumors reached $200mm^3$.

1.3 Isolation of immune cells

Immune cells in the spleen and tumors were isolated two days after the final treatment as previously described.[2] Cell counts were performed using 123count eBeads (ThermoFisher Scientific).

1.4 Flow cytometry and antibodies

Antibody labeling of cells for flow cytometry (1-10×10^6 immune cells) was performed using the BD Cytofix/Cytoperm Kit (BD Biosciences) according to the manufacturer's instructions. Antibodies and matched isotypes were obtained from the listed manufacturers. Flow cytometry (≥1×10^5 events) was performed on a BD LSRFortessa flow cytometer (Beckton Dickinson) and analyzed with FlowJo FACS Analysis Software v9.9.6 (Treestar). Cell populations were identified as listed (Supplemental Tables S1 and S2). Expression of phenotypic proteins was determined by subtracting the respective isotype, set between 1-5% of the population.

1.5 T cell restimulation

Isolated primary tumor immune cells were stimulated for 4 hours in the presence of 2μg/ml GolgiPlug (BD Biosciences) with nothing or 1μg/ml αCD3 (2C11, BD Biosciences) + 1μg/ml αCD28 (37.51, BD Biosciences). Frequencies of stimulated IFNγ^+ and/or TNFα^+ cells were calculated by subtracting the non-stimulated controls.

1.6 Antigen-specific CD8+ T cell responses

IFNγ responses to the vaccine antigen Twist were monitored using BD Mouse IFNγ ELISPOT set per the manufacturer's protocol. Briefly, 5×10^5 splenocytes were co-cultured for 24 hours with 10 μg/mL of HIVgag$_{197-205}$ (null), or a peptide pool spanning amino acids 141-178 of the murine Twist protein. Spot-forming cells (SFC) were quantified with an ImmunoSpot analyzer using the Smart Count ImmunoSpot software (Cellular Technology, Ltd.). SFC were calculated by subtracting the number of spots generated with null peptide from spots with peptide of interest.

1.7 Statistics

Statistical analyses were performed in Prism 7.0a (GraphPad Software). Unless otherwise stated, data presented in bar graphs or scatter plots were analyzed using one-way ANOVA with Tukey's multiple comparisons. Outliers were removed using the **Ro**bust regression and **Ou**tlier removal (ROUT) method. Statistical significance was set at $p <0.05$. *$p < 0.05$, **$p < 0.01$, ***$p < 0.001$, ****$p < 0.0001$.

2 References for Supplementary Material

1. Rovero S, Amici A, Di Carlo E, *et al.* DNA vaccination against rat her-2/Neu p185 more effectively inhibits carcinogenesis than transplantable carcinomas in transgenic BALB/c mice. J Immunol 2000;165(9):5133-42.

2. Knudson KM, Hicks KC, Alter S, *et al.* Mechanisms involved in IL-15 superagonist enhancement of anti-PD-L1 therapy. J Immunother Cancer 2019;7(1):82.

3 Supplementary Figures and Tables

Supplemental Table S1. Flow cytometry antibodies for identification of murine immune cell populations.

Antibody	Clone	Company
FoxP3	R16-715	BD Biosciences
CD62L	MEL-14	BD Biosciences
CD44	IM7	BD Biosciences
CD3e	2C11	BD Biosciences

IFNg	XMG1.2	BD Biosciences
TNFa	MPG-XT22	BD Biosciences
CD8a	53-6.7	ThermoFisher Scientific
FoxP3	FJK-16s	ThermoFisher Scientific
CD8b	53-5.8	Biolegend
CD45.2	104	Biolegend
CD4	RM4-4	Biolegend
CD4	RM4-5	Biolegend

Supplemental Table S2. Flow cytometry gating strategy used for identification of murine immune cell populations.

**Cell Population**	**Flow Cytometry Gating Strategy**
$CD8^+$ T Cells	Live/$CD45.2^+$/$CD3e^+$/$CD8a^+$
$CD4^+$ T Cells	Live/$CD45.2^+$/$CD3e^+$/$CD4^+$/$FoxP3^-$
$CD4^+$ T_{reg}	Live/$CD45.2^+$/$CD3e^+$/$CD4^+$/$FoxP3^+$

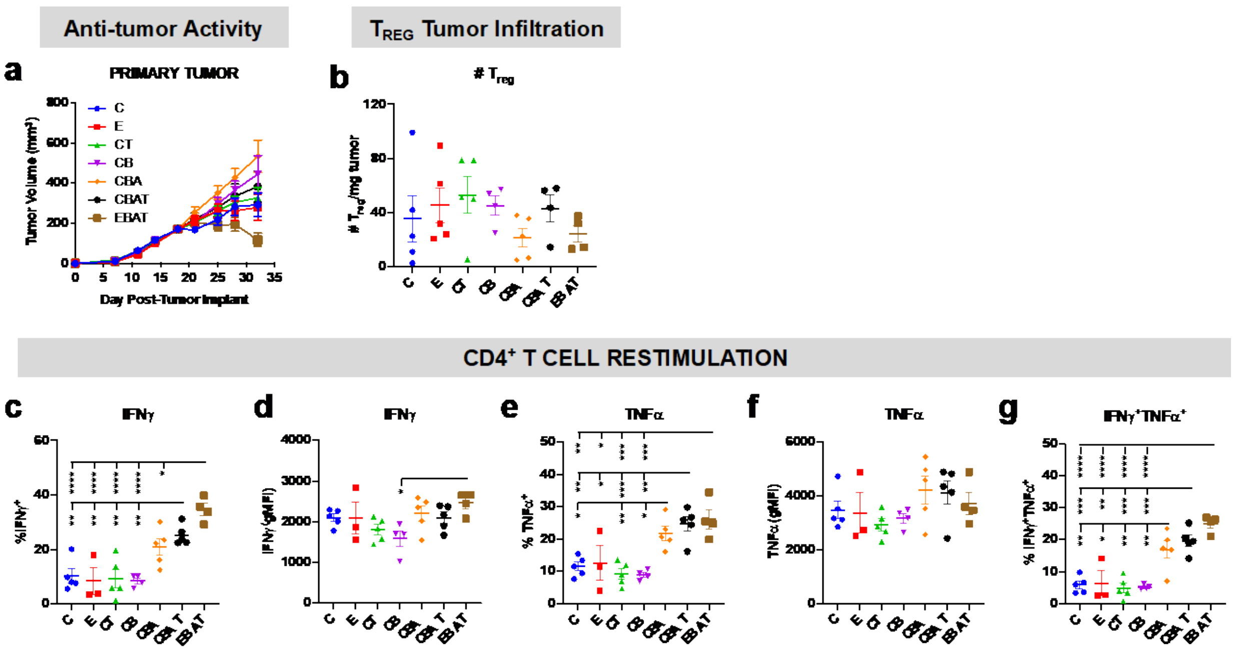

Supplemental Figure S1: Tumor Growth and Immune Correlates. TuBo breast tumor cells ($4x10^5$) were orthotopically implanted into the mammary fat pad of female Balb/c mice and treated with either control (C) or Entinostat (E) diet, alone or in combination with T-DM1 (T), Bintrafusp alfa (B), and/or Ad-TWIST (A) according to the schedule and doses in Figure 2. (a) Graphs show mean primary tumor growth curves ± S.E.M.. Data from one experiment, n=11-12 mice/group. (b-g) Two days after the last vaccination, tumor immune cells were isolated. Graph show the number of T_{reg} (b) in the tumor. (c-g) Tumor $CD4^+$ T cells were stimulated with αCD3 and αCD28 for 4 hours and cytokine production was analyzed by flow cytometry. Graphs show frequency of total $IFN\gamma^+$ (c), IFNγ production on a per cell basis (d), frequency of total $TNF\alpha^+$ (e), TNFα production on a per cell basis (f), and frequency of IFNγ/TNFα-double producing (g) $CD44^{hi}$ $CD4^+$ T cells. Data from 1 experiment, n=3-5 mice/group. All graphs show mean±SEM.
